# Oral health and oral health risk behaviour in children with and without externalising behaviour problems

**DOI:** 10.1007/s40368-018-0346-8

**Published:** 2018-05-15

**Authors:** M. Staberg, J. G. Norén, L. Gahnberg, A. Ghaderi, C. Kadesjö, A. Robertson

**Affiliations:** 1 Department of Pediatric Dentistry, Institute of Odontology, The Sahlgrenska Academy, University of Gothenburg, P.O. Box 450, 405 30 Gothenburg, Sweden; 2 Department of Behavioral and Community Dentistry, Institute of Odontology, The Sahlgrenska Academy, University of Gothenburg, Gothenburg, Sweden; 3 Department of Clinical Neuroscience, Karolinska Institutet, Stockholm, Sweden; 4 Gillberg Neuropsychiatry Centre, Institute of Neuroscience and Physiology, The Sahlgrenska Academy, University of Gothenburg, Gothenburg, Sweden

**Keywords:** Child behaviour, Conduct problems, Dental caries, Dental fear, Dental trauma, Disruptive behaviour disorder

## Abstract

**Aim:**

This was to study children with early detected externalising behaviour problems compared to matched controls regarding oral health, oral health risk behaviour and the parental evaluation of the child’s oral health and dental care.

**Methods:**

Children aged 10–13 years and with externalising behaviour problems, were compared to matched controls. Behavioural characteristics were based on the Strength and Difficulties Questionnaire. The children and their parents completed questionnaires regarding dental fear, tooth brushing, dietary habits and evaluation of oral health and dental care. Data on dental caries risk assessments, caries, behaviour management problems and dental trauma were obtained from dental files.

**Results:**

There were no differences in caries prevalence in children with early detected externalising behaviour problems, compared to controls. However, the former group consumed more sweet drinks when thirsty and brushed their teeth fewer than twice daily; they also had more dental trauma in both dentitions and a higher risk range for dental fear, compared to controls.

**Conclusions:**

This study points out potential oral health risk factors in children with early-detected externalising behaviour problems. Although no difference in caries prevalence was observed, externalising behaviour may affect oral health. Therefore, dental professionals should support the families and the children to preserve dental health by offering increased prophylactic measures. There were no differences between children with externalising behaviour problems, compared with controls, regarding the parent evaluation of their child’s dental health. However, more parents in the study group evaluated the dental care as poor or not functioning.

## Introduction

A considerable number of children and adolescents suffer from emotional and behavioural problems. According to a British review the prevalence of having signs of significant problem behaviour is between 10 and 20% in children and adolescents (Ogundele 2018). Childhood behaviour problems, such as hostile aggression and hyperactivity, are undesired due to norms of conventional society and defined as behaviour that is socially a problem. Externalising behaviour problems (EBP) include attention deficit hyperactivity disorder (ADHD) problems (inattention, hyperactivity/impulsivity), as well as disruptive, oppositional, aggressive, and conduct disorder behaviour (Bloomquist and Schnell 2002).

Externalising behaviour in children has been shown to influence both dental care and oral health (Staberg et al. 2014a, b). An oral health risk behaviour can be expressed as a child brushing its teeth less than twice a day, and consuming more sweets and sweetened drinks several times a day.

Therefore, it is important to establish good routines in childhood, to promote and improve oral health. Good oral health habits can continue throughout adulthood, giving a lifelong protection from dental diseases (Loe 2000; Aunger 2007).

In children with ADHD, the frequent consumption of sugar can be difficult for the parents to deal with, and sometimes, the oral hygiene/tooth brushing is neglected (Staberg et al. 2014b). Among children with externalising behaviour problems, those with an elevated caries risk have been shown to have more impulsivity and conduct problems, compared to children with low caries risk (Staberg et al. 2016).

Children with traumatic dental injuries (TDI) have more hyperactive symptoms than children without dental trauma (Herguner et al. 2015). The frequency of dental injuries in children with ADHD peaks at the age of 10–12 years, with the main causes of dental injuries being falls, collisions with objects, violence and traffic accidents (Avsar et al. 2009).

A Swedish review article has found a relationship between dental fear and children with externalising problems (Klingberg and Broberg 2007). Dental anxiety and behaviour management problems are higher in children with ODD/ ADHD, than in children without ODD/ADHD (Aminabadi et al. 2016), and may delay or prevent dental treatment.

All children in Sweden are assessed for caries risk at their regular dental examinations, with the outcome used for caries preventive planning for those children who need it the most (Twetman et al. 2013). Therefore, it is important to evaluate if, and in what way, oral health differs between children with early-detected externalising problems, and matched controls. In cases where it is indicated, this information can be used to design and implement early, effective interventions and provide active help for children with externalising problems, and their parents.

The aim of the present paper was to study children with early-detected externalising behaviour problems, compared to matched controls, regarding oral health, oral health risk behaviour and the parent’s evaluation of the child’s oral health and dental care.

## Hypothesis

Children with externalising behaviour problems have a risk behaviour influencing caries, dental trauma, dental fear, and poorer oral health routines (e.g., less frequent tooth brushing habits and more frequent cariogenic dietary habits compared to controls).

## Subjects and methods

### Study group

The study group was comprised of 194 families with children (10–13 years of age), whose parents participated in parent management training (PMT) programs, evaluating early intervention for children with externalising behaviour problems, previously described in detail (Staberg et al. 2016).

**Fig. 1:**
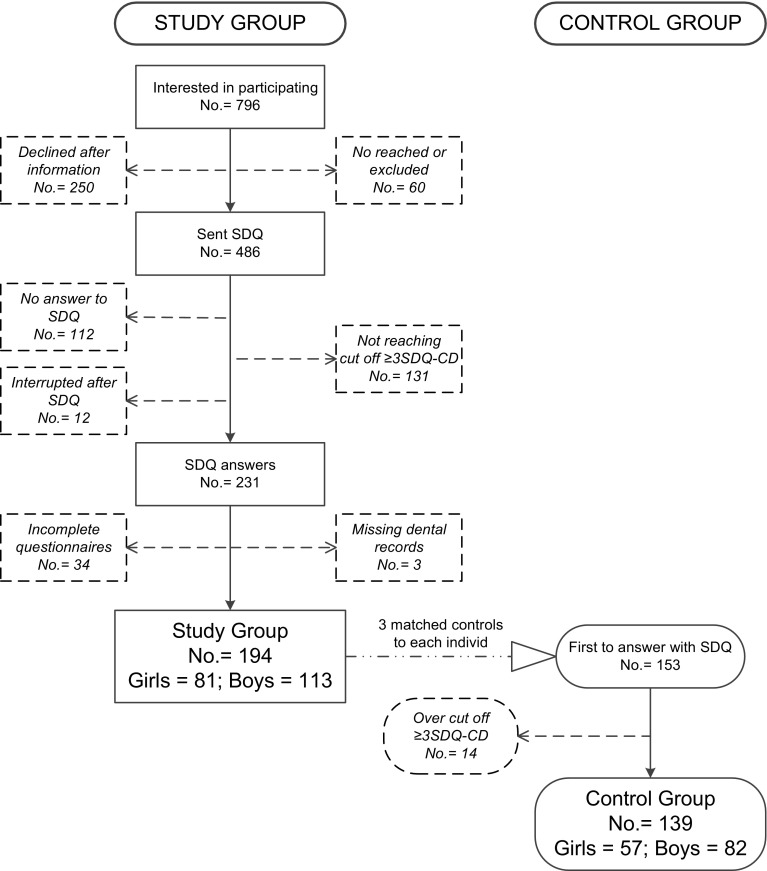
Flow chart describing the recruitement of patients to the study group and to the control group, respectively. *SDQ* Strengths and Difficulties Questionnaire, *SDQ-CD* Strengths and Difficulties Questionnaire conduct problems

Briefly, 796 families, who experienced some degree of externalising behaviour problem with their child (e.g., parents with children in conflict with peers, parents or other adults, protesting against demands, often restless, having friends with bad influence or having been involved in vandalism, shoplifting or truancy) responded to advertisements about participating in the study. After obtaining written informed consent, the parents were asked to fill out the Strengths and Difficulties Questionnaire (SDQ) (Goodman 1997). Children below the cut-off point, the criteria for clinically relevant problems (less than three points on the conduct problem subscale of the SDQ), and children with autism, obsessive compulsive disorder or ongoing psychiatric treatment, were excluded. Finally, 231 families entered the study; 3 children were excluded due to missing dental records, and 34 children were excluded due to missing questionnaire and/or dental data, resulting in a total number of 194 children (113 boys, 81 girls). A flow chart illustrating the recruitment process and dropouts is presented in Fig. 1.

### Control group

For each child in the study group, three possible matched controls, with the same age, gender, dental clinic and socioeconomic area (residential address), were identified. The first one, of the three matched controls, accepting the invitation was selected. It was possible to recruit 194 children into the study group and 153 into the control group (63 girls and 90 boys). All parents were asked to fill out the same questionnaire (SDQ) as the study group. Children with a value ≥ 3 or more on the conduct problem subscale of the SDQ, (six girls, eight boys), were excluded to ensure a control group without externalising behaviour problems, resulting in 139 controls (57 girls, 82 boys) (Fig. 1).

### Instruments

#### Background information questionnaires

The parents were asked to provide background information through a questionnaire, regarding dental care and the parent’s evaluation of their child’s oral health. The child responded to a questionnaire regarding dental fear, tooth brushing frequency, and dietary habits.

#### The Strengths and Difficulties Questionnaire (SDQ)

The Strengths and Difficulties Questionnaire (Goodman 1997) is a frequently used screening instrument for child and adolescent mental health, throughout the world, with good psychometric properties (Goodman 2001). The parental version of the SDQ for children 4–16 years, used in this study, can be completed within a few minutes and is validated for Swedish conditions (Smedje et al. 1999).

The SDQ symptom scales contain 25 items divided into five subscales, namely, Emotional Symptoms, Conduct Problems, Hyperactivity-Inattention, Peer Problems, and Prosocial Behaviour. A 3-point Likert scale is employed to indicate how each attribute applies to the target child (0 = not true; 1 = somewhat true; 2 = certainly true). All subscales, with the exception of prosocial behaviour, are summed together to a total difficulties score. A high score on the Prosocial Behaviour subscale indicates a strength, while high scores on the other four subscales indicate difficulties.

#### Dental fear (CFSS-DS)

The Dental Subscale of the Children’s Fear Survey Schedule (CFSS-DS) is a well-known instrument for assessing dental fear in children, initially presented by Cuthbert and Melamed (1982). The CFSS-DS consists of 15 items, related to various aspects of dental treatment. Each item can be scored on a 5-point scale from 1 (not afraid) to 5 (very afraid). Total scores range from 15 to 75.

The cut-off score of 38 or higher on the CFSS-DS has been commonly used to define dental fear, irrespective of age, gender, and informant. In the present study, the cut-off score was set to ≥ 32 points, indicating “borderline” or “risk for dental fear”, which has been used in previous studies (ten Berge et al. 2002; Fagerstad et al. 2015). Some children have no, or very limited, experience of invasive dental treatment and are therefore unable to answer all 15 questions in the survey on the CFSS-DS. Where responses to one or a maximum of three survey questions were missing then an average score was calculated. That score was used, thereby, so that a total of CFSS-DS could still be established.

### Dental records

Data from dental records regarding caries in the primary teeth (deft, 12 teeth canine, first and second primary molars), caries in the permanent teeth (DMFT) and initial caries in first permanent molars, were compiled. Since children are growing individuals with different dental stages, ages, and number of teeth, caries in the first permanent molar was chosen as an expression for the caries situation.

All Swedish children are assessed for caries risk at their dental examinations. Data regarding caries risk was compiled from the dental file system used, by the Public Dental Service in the Region of Västra Götaland. The caries risk assessment is set by a combination of the computerised algorithm-based system R2 (Andas and Hakeberg 2014), and a clinical assessment made by each child’s regular dentist, according to regional standardised guidelines by the Region of Västra Götaland. Those guidelines can be obtained by contacting the corresponding author.

The caries activity, based on new caries lesions and caries progression, is estimated in combination with modifying factors such as diet, fluoride, oral hygiene, previous caries experience, age, and medical risk recorded. The R2 system finally defines the caries risk as low, intermediate, or high. In order to identify children at risk, the caries risk data were dichotomised to low and elevated caries risk. The intermediate and high caries risk group together, formed the elevated caries risk group.

Data regarding dental trauma in the primary and permanent teeth, behaviour management problems (BMP), defined as notes in the dental records, clearly expressing severe disruptive behaviours, were also collected from dental files. In this study all dental files have been reviewed and read through from the very first dental visit.

### Statistical analysis

Statistical analysis was performed using the statistical software R (GNU General Public License, Free Software Foundation, Inc., Boston, USA) and the Statistical Package for Social Sciences (SPSS version 21). Bonferroni–Holm corrected p-values were calculated by the multitest procedure in SAS Version 9.3 (SAS institute. Ink, Cary, NC, USA).

A logistic regression was used to assess the association between children with externalising behaviour problems and dental caries, traumatic dental injuries, oral health risk factors, dental fear and parental evaluation of dental care, and the child’s oral health, compared to controls. Data were adjusted for age and gender. The results were expressed as odds ratio (OR) with a 95% confidence interval. For multiple interferences, the significance level was adjusted according to the Bonferroni–Holm method and in the results, both un-adjusted and adjusted values are presented.

#### Ethical considerations

The study was approved by the Ethical Committee in Uppsala (dnr 2010/119). All families participating in the project were given written information. Written consent from the participating families was received, in order to acquire access to their child’s dental records.

## Results

In order to make the presentation of the results more explicit, the results are shown in four different tables, including un-adjusted and adjusted p-values.

### Study group vs. control group

#### Gender and year of birth

The distribution of age and gender in the study group and the control group were approximately similar. The mean age in the study group was 11.7 years (SD 1.6) and the corresponding values in the control group were 11.6 years (SD 1.7).

### Caries and caries risk assessment (R2)

#### Caries

No statistical significant difference was found regarding caries in the primary and permanent teeth, and caries in the primary and/or permanent dentition, and number of decayed, missing/filled first permanent molars, including initial caries between the two groups.

Upon entering the study, 28.9% of the children in the study group had filled or decayed first permanent molars, compared to 18.7% of the controls. The difference was statistically significant in the logistic regression analysis (*p* = 0.038), however, after Bonferroni–Holm correction (BH-c), the difference was not significant. The OR for DMFT > 0 was 1.78 (Table 1).

**Table 1:** The upper part of the table shows the number of children with primary dental caries and permanent dental caries, caries in the primary and/or permanent dentitions, number of decayed/missing/filled first permanent molars in the study and control groups, the distribution in low and elevated caries risk groups, respectively, when entering the study

	Study group	Control group	Total
	n (%)	n (%)	n (%)
Caries			
Caries in primary teeth			
deft = 0	137 (70.6)	108 (77.7)	245 (73.6)
deft > 0	57 (29.4)	31 (22.3)	88 (26.4)
Caries in permanent teeth			
DMFT = 0	130 (76.0)	102 (73.4)	232 (69.7)
DMFT > 0	64 (33.0)	37 (26.6)	101 (30.3)
Caries in primary and/or permanent dentition			
deft and DMFT = 0	96 (49.5)	82 (59.0)	178 (53.5)
deft and DMFT > 0	98 (50.5)	57 (41.0)	155 (46.5)
Number of decayed/missing/filled first permanent molars			
DMFT = 0	138 (71.1)	113 (81.3)	251 (75.4)
DMFT > 0	56 (28.9)	26 (18.7)	82 (24.6)
Number of decayed/missing/filled first permanent molars including initial caries			
DMFTi = 0	111 (57.2)	93 (66.9)	204 (61.3)
DMFTi > 0	83 (42.8)	46 (33.1)	129 (38.7)
Caries risk assessment			
Low risk	138 (71.1)	114 (82.0)	252 (75.7)
Elevated risk	56 (28.9)	25 (18.0)	81 (24.3)

	n	OR	CI	p log reg	p log reg B-H
Caries in primary teeth	333	1.45	0.88–2.42	n.s	n.s
Caries in permanent teeth	333	1.35	0.82–2.22	n.s	n.s
Caries prim and/or perm dent	333	1.46	0.94–2.28	n.s	n.s
DMFT	333	1.78	1.04–3.09	0.038	n.s
DMFTi	333	1.51	0.95–2.43	n.s	n.s
Caries risk assessment	333	2.42	0.98–6.86	n.s	n.s

Percentage within brackets (*Deft* decayed/extracted/filled primary teeth, *DMFT* decayed/missing/filled first permanent molars, *DMFTi* decayed/missing/filled first permanent molars and initial caries)

The lower part of the table shows the results from the logistic regression [*n* number, *n.s*. non-significant, *OR* odds ratio, CI confidence interval (95%), *p log reg* p-value logistic regression, *p log reg B–H* p-value logistic regression with Bonferroni–Holm correction]

#### Caries risk assessment (R2)

In the study group, 28.9% of the children had an elevated caries risk, compared to 18% in the control group, and the difference was not statistically significant. The OR for elevated caries risk was 2.42 (Table 1).

### Oral health behaviour

#### Tooth brushing

More children with externalising behaviour brushed their teeth less than twice a day, and when compared to the controls, the difference was statistically significant (*p* = 0.0007 after BH-c *p* = 0.01) (Table 2). The OR for tooth brushing less than twice a day was 2.80.

**Table 2:** The upper part of the table shows the frequencies of the risk factors connected to oral health behaviour in children with externalising behaviour problems compared to controls

	Study group	Control group	Total
	n (%)	n (%)	n (%)
Tooth brushing			
TB < 2 times/day	56 (28.9)	18 (12.9)	74 (22.2)
TB ≥ 2 times/day	138 (71.1)	121 (87.1)	259 (77.8)
Drink when thirsty			
Water/milk	144 (74.2)	125 (89.9)	269 (80.8)
Other than water/milk	50 (25.8)	14 (10.1)	64 (19.2)
Sweet /soft drinks at meals			
Never, seldom, 1/week	116 (59.8)	98 (70.5)	214 (64.3)
Several times /week/daily	78 (40.2)	41 (29.5)	119 (35.7)
Sweets			
Never, seldom, 1/week	127 (65.5)	103 (74.1)	230 (69.1)
Several times /week/daily	67 (34.5)	36 (25.9)	103 (30.9)
Cakes, buns biscuits			
Never, seldom, 1/week	163 (84.0)	109 (78.4)	272 (81.7)
Several times /week/daily	31 (16.0)	30 (21.6)	61 (18.3)

	n	OR	CI	p log reg	p log reg B-H
Tooth brushing	333	2.80	1.58–5.19	0.0007	0.010
Drink when thirsty	333	3.13	1.68–6.19	0.0005	0.009
Sweet/soft drinks at meals	333	1.61	1.02–2.58	0.0447	n.s
Sweets	333	1.50	0.93–2.45	n.s	n.s
Cakes, buns, biscuits	333	0.69	0.39–1.21	n.s	n.s

Percentage within brackets

The lower part of the table shows the results from the logistic regression [*n* number, *n.s*. non-significant, *OR* odds ratio, *CI* confidence interval (95%), *p log reg* p-value logistic regression, *p log reg B–H* p-value logistic regression with Bonferroni–Holm correction]

#### Drinking when thirsty

Children with externalising behaviour preferred drinks other than water or milk, more often when thirsty, compared to the controls. The logistic regression analysis showed a statistically significant difference (*p* = 0.0005; after BH-c *p* = 0.009) (Table 2). The OR for preferring other beverages than water or milk when thirsty was 3.13.

#### Sweet/soft drinks at meals

In the study group, 40.2% of the children frequently (several times/week/daily) drank sweetened drinks at meals, compared to 29.5% in the control group, however, the difference was not statistically significant. The OR for drinking sweetened/soft drinks at meals several times a week/daily was 1.62 (Table 2).

#### Sweets

Children with externalising behaviour more often consumed sweets several times per week or daily compared to the controls (34.5 vs. 25.9%), but the difference was not statistically significant. The OR for consuming sweets several times /week/daily was 1.50 (Table 2).

#### Cakes, buns, biscuits

No differences were found regarding the consumption of cakes, buns and biscuits between the two groups (Table 2).

### Traumatic dental injuries

There were more children with externalising behaviour who had traumatic dental injuries (TDI) in both dentitions, compared to the controls (51.5 and 30.2%, respectively). The logistic regression showed a statistically significant difference (*p* < 0.0001; after BH-c *p* = 0.002; OR 2.47) (Table 3).

**Table 3:** The upper part of the table shows the frequencies of traumatic dental injuries (TDI) in the primary and permanent dentitions in children with externalising behaviour problems compared to controls

	Study group	Control group	Total
	n (%)	n (%)	n (%)
TDI both dentitions			
No TDI	94 (48.5)	97 (69.8)	191 (57.4)
TDI	100 (51.5)	42 (30.2)	142 (42.6)
TDI primary dentition			
No TDI	132 (68.0)	116 (83.5)	248 (74.5)
TDI	62 (32.0)	23 (16.5)	85 (25.5)
TDI permanent dentition			
No TDI	134 (69.1)	114 (82.0)	248 (74.5)
TDI	60 (30.9)	25 (18.0)	85 (25.5)

	n	OR	CI	p log reg	p log reg B-H
TDI both dentitions	333	2.47	1.57–3.93	0.0001	0.002
TDI primary dentition	333	2.42	1.42–4.22	0.0014	0.020
TDI permanent dentition	333	2.04	1.21–3.52	0.0082	n.s

Percentage within brackets

The lower part of the table shows the results from the logistic regression [*n* number, n.s. non-significant, *OR* odds ratio, CI confidence interval (95%), *p log reg* p-value logistic regression, *p log reg B–H* p-value logistic regression with Bonferroni–Holm correction]

TDI in the primary dentition was statistically significantly more common among the externalising children, compared to the controls (32 vs.16.5%, *p* = 0.0014; after BH-c *p* < 0.02; OR 2.42). In the permanent dentition, TDI was significantly more common among the externalising children (30.9 vs. 18%; *p* = 0.008; OR 2.04), however, the difference was non-significant after BH-c (Table 3).

### Behaviour management problems (BMP)

The frequency of BMP was 10.3% in the study group and 2.2% in the control group, and the difference was statistically significant (*p* < 0.009; after BH-c non-significant; OR 5.25).

## Risk for dental fear (CFSS-DS)

There were 10 children in the study group with dental fear (CFSS-DS ≥ 38), however, none in the control group reached a value of CFSS-DS ≥ 38. The mean value for the CFSS-DS score in the study group was 24.07 (SD 7.403), and in the control group 20.16 (SD 4.677).

In the study group and the control group, 15.5% and 2.2% of the children had a CFSS-DS value ≥ 32, respectively, and were thus classified as having a higher risk for dental fear. The difference was statistically significant (*p* = 0.0005; after BH-c *p* = 0.009; OR 8.61). No correlation was found between risk for dental fear and TDI.

### Parental evaluation of dental care and dental health

There was no statistical difference between children with externalising behaviour problems, compared to controls, regarding the parent’s evaluation of their child’s dental health. The OR value was 2.34 (Table 4). There were more parents in the study group evaluating the dental care as poor or not functioning (*p* = 0.03; after BH-c non-significant; OR 4.05) (Table 4).

**Table 4:** The upper part of the table shows the frequencies of behaviour management problems (BMP), risk for dental fear according to Children’s Fear Survey Schedule (CFSS-DS) and parental evaluation of dental care and the child’s dental health in children with externalising behaviour problems compared to controls

	Study group	Control group	Total
	n (%)	n (%)	n (%)
Behaviour management problems			
No BMP	174 (89.7)	136 (97.8)	310 (93.1)
BMP	20 (10.3)	3 (2.2)	23 (6.9)
Risk for dental fear			
CFSS-DS < 32p	164 (84.5)	135 (97.1)	299 (90.0)
CFSS-DS ≥ 32p	30 (15.5)	3 (2.2)	33 (9.9)
Dental health (parental evaluation)			
Very good	95 (52.5)	97 (69.8)	192 (60.0)
Good/poor	86 (47.5)	42 (30.2)	128 (40.0)
Dental care (parental evaluation)			
Well-functioning	178 (91.8)	136 (97.8)	314 (94.3)
Poor	16 (8.2)	3 (2.2)	19 (5.7)

	n	OR	CI	p log reg	p log reg B-H
BMP	333	5.25	1.75–22.63	0.0086	n.s
Risk for dental fear	332	8.61	2.96–36.60	0.0005	0.0089
Evaluation by parents					
Dental health	320	2.34	0.68–10.71	n.s	n.s
Dental care	333	4.05	1.31–17.66	0.0289	n.s

Percentage within brackets

The lower part of the table shows the results from the logistic regression [*n* number, *n.s*. non-significant, *OR* odds ratio, *CI* confidence interval (95%), *p log reg* p-value logistic regression, *p log reg B–H* p-value logistic regression with Bonferroni–Holm correction]

## Discussion

This study has shown that the caries prevalence was not higher in children with early-detected externalising behaviour problems, compared to the controls. However, it was more common that these children brushed their teeth fewer than twice a day, and consumed more sweetened drinks. These individual risk factors might lead to a future increased risk of being in the elevated caries risk group. Furthermore, these children had more dental trauma in both dentitions, and a higher risk range for dental fear, compared to the controls.

A strength of the present study was the selection criteria of including families from different socio-economic areas, and the use of the validated instruments, SDQ and CFSS-DS. In this study, the Bonferroni–Holm correction was used, but the unadjusted values are also presented, adding valuable information.

Children with early-detected externalising behaviour problems had fared well in terms of caries. There was no significant difference regarding caries, compared to the controls, which is in accordance with a previous study of young children (aged 3–8 years) (Lorber et al. 2014). In a study of children with ADHD at age 13 years, the caries prevalence was not higher compared to controls (Blomqvist et al. 2007). However, teenagers (aged 17 years) with ADHD had a higher prevalence of caries, compared to the controls (Blomqvist et al. 2011).

The OR value of 2.42 in the caries risk assessment found in the present study indicated a need for special attention regarding caries in children with externalising behaviour. Since the individual risk factors may lead to a higher future caries activity during adolescence, these children should belong to the elevated caries risk group. This study has pointed out potential risk factors in children with early-detected externalising behaviour problems. This means that the dental professionals have great opportunities to assist and support the families by offering an increased number of contact times and prophylactic measures.

Poor oral hygiene, tooth brushing and consumption of sweetened beverages, identified in the study group compared to controls, may increase the risk for future caries development. This is in agreement with the results of a Norwegian study in pre-school children (Wigen and Wang 2015). Previous studies have shown an association between ADHD and less frequent tooth brushing, irregular eating times, unhealthy food, and the increased consumption of soft drinks/sweetened beverages (Ptacek et al. 2014; Staberg et al. 2014b).

Oral health and daily tooth brushing routines from the parents to the child are usually established early in life and are resistant to changes (Aunger 2007). Healthy eating requires planning, organisation and self-regulation, which may be more difficult for teenagers with externalising behaviour problems. As a child grows older and becomes more independent, the risk factors may increase when supervision from the parent’s decreases.

In this study, the frequency of traumatic dental injuries was higher among children with externalising behaviour problems, compared to the controls. The prevalence of dental trauma in both dentitions, 51.5%, was higher than the 42.9% found among 11–13-year-old in a previous Swedish study on dental trauma (Oldin et al. 2015). Therefore problem behaviour can be an additional risk factor for traumatic dental injuries (TDI), which is in line with a previous study (Oldin et al. 2015). Furthermore, the children at risk for dental fear, CFSS-DS ≥ 32, had not been exposed to more dental trauma compared to those without dental trauma.

The children in the study group had to have a value of 3 or more on the SDQ-CD scale to be regarded as having a clinical relevant behaviour problem. Due to the child’s impulsivity and conduct problems, this risk for “acting out” behaviour may lead to more arguments with friends and intentional or unintentional injuries.

The child’s activities and the environment, e.g., the child’s sociability, may be another determining risk factor for TDI, which is in agreement with a recently presented Swedish study (Oldin et al. 2015). Impulsivity and attention-related problems, associated with externalising behaviour problems, may influence the child’s inhibition systems and limit the child’s risk assessment, leading to activities without thinking of consequences and thereby, increasing the risk for dental trauma.

Studies from the UK have established a relationship between emotional disorders and unintentional injuries, where children with disruptive behaviour had an increased risk for injuries, as a result of their hyperactivity and emotionality (Lalloo et al. 2003; Rowe et al. 2004). Due to the trauma risk, interceptive orthodontic treatment in children with externalising behaviour and a large incisal overjet could be a good preventive measure, since the incisal overjet has been shown to be a risk factor for TDI (Forsberg and Tedestam 1993).

A high OR value for behaviour management problems was found among the children with externalising behaviour problems in this study. The connection between BMP and externalising behaviour are in line with earlier findings in a Swedish study (Blomqvist et al. 2004). One factor that can explain the behaviour management problems is the age of the child, since BMP has been shown to decline with age (Klingberg et al. 1994). The BMP could also possibly be explained by the dental team’s inexperience of treating externalising children (Staberg et al. 2014a). Dental teams, who are able to develop warm and supportive relationships with these children, have the potential to create a well-functioning and positive dental experience, and reduce the risk for a negative oral health outcome, which has been shown in the present study, where the parents were satisfied with the dental care.

## Conclusions

This study has pointed out potential oral health risk factors in children with early-detected externalising behaviour problems, compared to a matched control group. Although no difference in caries prevalence was observed, externalising behaviour may affect oral health, caries, and dental trauma, and may increase the risk for dental fear. By paying attention to the child’s behaviour and listening to the parents during the dental visit, the dental teams may be able to identify externalising children. This means the dental professionals have great opportunities to assist and support the families by offering an increased number of contact times and prophylactic measures. In this way, dental care may preserve the dental health of the children. There were no differences between children with externalising behaviour problems, compared to the controls, regarding the parent’s evaluation of their child’s dental health. However, in the study group, more parents evaluated the dental care as poor or not functioning.

## Clinical implications

This study has shown that externalising behaviour ought to be added to the repertoire of factors considered for caries risk evaluation. Since the parents evaluated the dental care as poor or not functioning, the dental professionals should consider this fact when treating and planning dental care. Furthermore, externalising children with a large overjet should be offered interceptive orthodontic treatment.

The collaboration in prevention between the Public Dental Service, the school, and the social services, described in this study, is unique for Swedish conditions.

If the regular dental service identifies children with externalising behaviour problems, this could be valuable for their oral health, and in addition, may initiate a contact between the family and the social services for support and help, such as participating in parent management training programmes.
